# Computational Testing for Automated Preprocessing 2: Practical Demonstration of a System for Scientific Data-Processing Workflow Management for High-Volume EEG

**DOI:** 10.3389/fnins.2018.00236

**Published:** 2018-04-10

**Authors:** Benjamin U. Cowley, Jussi Korpela

**Affiliations:** ^1^Cognitive Science, Department of Digital Humanities, University of Helsinki, Helsinki, Finland; ^2^Cognitive Brain Research Unit, Department of Psychology and Logopedics, University of Helsinki, Helsinki, Finland; ^3^Digitalization, Finnish Institute of Occupational Health, Helsinki, Finland

**Keywords:** EEG, electroencephalography, EEGLAB, scientific workflow system, workflow management, computational testing, automated preprocessing, parameter sweep

## Abstract

Existing tools for the preprocessing of EEG data provide a large choice of methods to suitably prepare and analyse a given dataset. Yet it remains a challenge for the average user to integrate methods for batch processing of the increasingly large datasets of modern research, and compare methods to choose an optimal approach across the many possible parameter configurations. Additionally, many tools still require a high degree of manual decision making for, e.g., the classification of artifacts in channels, epochs or segments. This introduces extra subjectivity, is slow, and is not reproducible. Batching and well-designed automation can help to regularize EEG preprocessing, and thus reduce human effort, subjectivity, and consequent error. The Computational Testing for Automated Preprocessing (CTAP) toolbox facilitates: (i) batch processing that is easy for experts and novices alike; (ii) testing and comparison of preprocessing methods. Here we demonstrate the application of CTAP to high-resolution EEG data in three modes of use. First, a linear processing pipeline with mostly default parameters illustrates ease-of-use for naive users. Second, a branching pipeline illustrates CTAP's support for comparison of competing methods. Third, a pipeline with built-in parameter-sweeping illustrates CTAP's capability to support data-driven method parameterization. CTAP extends the existing functions and data structure from the well-known EEGLAB toolbox, based on Matlab, and produces extensive quality control outputs. CTAP is available under MIT open-source licence from https://github.com/bwrc/ctap.

## 1. Introduction

Recording electroencephalography (EEG) data has become more affordable, scalable, and feasible in disparate conditions inside and outside the lab (Cowley et al., 2016, pp. 50–66), with research- and consumer-grade devices (Badcock et al., 2013). Methods and computing power to handle EEG datasets have also grown in complexity and power. It has consequentially become more and more important to manage the *scientific data-processing workflow* of recording EEG, to achieve best results.

In this regard, EEG research follows a similar trend to other data-intensive disciplines, e.g., bioinformatics (Leipzig, 2016), such that it requires a scientific workflow management system (SWMS) to give standardized, comparable results at scale. The needs of such an SWMS include:

1. enabling basic features for management of analysis pipelines,
2. enabling comparison of outputs between unrelated recording setups/analysis pipelines^1^,
3. reducing or removing reliance on trial and error for parameter optimization (Holl et al., 2014).

This paper describes a system for managing the pre-processing workflow for EEG recordings: implemented as the Computational Testing for Automated Preprocessing (CTAP) toolbox. CTAP is implemented for Matlab 2016b and above, based on the data-specification and functions from the popular EEGLAB toolbox (Delorme et al., 2011). The basic features of CTAP have previously been described in Cowley et al. (2017); therefore here we have a more practical focus on illustrating the usage of CTAP.

This paper describes three functional analysis pipelines, with separate Methods and Results subsections for each one, demonstrating CTAP's approach to each need 1–3 above. They are available from the CTAP repository at https://github.com/bwrc/ctap, as follows:

- ctap/templates/Frontiers_manuscript_examples/runctap_manu2_basic.m
- ctap/templates/Frontiers_manuscript_examples/runctap_manu2_branch.m
- ctap/templates/Frontiers_manuscript_examples/runctap_manu2_hydra.m

Each pipe processes EEG data obtained from a server hosted by the Schwartz Centre for Computational Neuroscience (SCCN), University of California San Diego. The final output of each pipe is a simple event-related potential (ERP) visualization of the conditions recorded in the dataset; any more complete analysis is assumed to depend on the user's own research question.

First, the *management of analyses* is illustrated with a basic linear pipeline, which shows the core CTAP structure of processing steps, quality control outputs, and usage options that help with processing management.

Second, *comparison of outputs* is illustrated with a branching pipeline. A branching tree structure of pipes enables users to extend the core functionality into a configuration that can compare competing processing approaches, while remaining a single project.

Third, *parameter optimization* is illustrated with a pipeline that utilizes repeated analyses of a given parameter range to discover the best performing value. By embedding a parameter optimization step in their pipe, users can go from testing a single parameter value to sweeping a range or set of values, extending the capability of CTAP to find an optimal analysis approach with controlled and tidy workflow management. The so-called HYDRA method (standing for Handler for sYnthetic Data Repeated Analysis), is still under development, yet to be published but available in the development branch of CTAP repository.

Note that there is considerable overlap between each way of using CTAP, and the three pipelines above focus on distinct themes merely for clarity. The ultimate use of CTAP is envisaged as a branching, parameter optimizing analysis manager, integrating all three themes.

Existing SWMSs tend to operate at a larger and more general scale than CTAP, targeting whole disciplines rather than just a single type of data (Curcin and Ghanem, 2008). Such systems allow automation of the repetitive cycle of configuring data for analysis, launching computations, and managing storage of results (Deelman et al., 2009). SWMSs thus aim to let scientists focus on research, not computation management. Already almost 10 years ago, reviews attempted to create a taxonomy of SWMSs (Curcin and Ghanem, 2008; Deelman et al., 2009), describing most workflow platforms and languages as originating in a particular application domain. On the other hand, the workflows themselves can usually conform to a finite set of patterns or “motifs” (Garijo et al., 2014). This has driven further research on interoperability (Terstyanszky et al., 2014) and search (Starlinger et al., 2016) to help integrate SWMSs; while also implying that separate SWMSs can retain a more intra-disciplinary focus (supported by recent reviews Liu et al., 2015; Leipzig, 2016). A particularly interesting development for EEG-researchers is the investigation of optimization of workflow computation costs (Kougka and Gounaris, 2014), and/or optimization of factors within workflows, e.g., process parameters (Holl et al., 2014).

There are several criteria that an SWMS should meet. Saxena and Dubey (2011) specified four:

1. provide facilities for specifying workflows: inputs/outputs, intermediate steps, and parameters,
2. provide facilities for managing data provenance,
3. provide facilities to monitor the progress of the workflow, include facilities to detect anomalies, isolate faults and provide recovery actions, and
4. manage the execution of the workflow based on specified parameters/configurations.

For ease of reference, we summarize these criteria as (1) *replicable*, (2) *traceable*, (3) *self-monitoring*, (4) *configurable/scalable*. To these four criteria we add a fifth, based on recent advances in the literature (Holl et al., 2014; Kougka and Gounaris, 2014): (5) *data-driven*, i.e., providing facilities to adapt/optimize processes with respect to input variability.

We have previously described how CTAP relates to the state of the art for processing EEG, in a recently published open access article (Cowley et al., 2017). This discussion can be summarized as such: CTAP aims to address a niche need in the EEGLAB ecosystem, rather than aim to compete with existing standalone solutions. More recently, a number of contributions have been made following a similar agenda as CTAP, several gathered in this Research Topic. Frömer et al. (2018) present an EEG processing pipeline based on EEGLAB (and other) functions, which aims to support single-trial processing for robust statistical analysis. Gabard-Durnam et al. (2018) describe an automated EEG processing pipeline aimed at high-artifact data. In the realm of magnetoencephalography processing, Andersen (2018) details a pipeline based on the popular MNE-Python software, which aims at reproducible group analysis. At the time of writing, these tools seem to have somewhat similar philosophy yet different motivations. It seems likely that the literature is experiencing a “zeitgeist” of developing support for workflows and automation; thus all these contributions represent functionality that could complement each other, if further developed.

Based on the described state of the art and established SWMS criteria, CTAP can be considered as a highly specific form of SWMS, integrating workflow management and parameter optimization together with the rich existing body of methods and tools in the EEGLAB ecosystem.

In this paper, we will discuss CTAP usage, both as defined in three example pipelines, and also more subtle usage considerations such as how data storage affects use. Thus we focus on how CTAP *is used*, and leave to other sources (Cowley et al., 2017) the question of how CTAP *works*. We focus on each pipeline in turn, describing how each one works in the Methods subsections, and their outcomes in the Results subsections. In the Discussion, we describe how each SWMS criterion is met via one or more of the three usage scenarios of CTAP, and point out further capabilities, limitations, and future work.

## 2. Methods

In the text below, code elements (including script names) are listed in courier; functions are marked by “()”; scripts are marked by “.m.”

### 2.1. Materials

Continuous EEG data was obtained from the database of HeadIT (Human Electrophysiology, Anatomic Data and Integrated Tools Resource). The data set is freely and permanently available, is described file-by-file at the source website^2^, and was chosen due to its simple and classic oddball trial structure. The protocol was an auditory two-choice response task with an ignored feature difference, i.e., participants categorized tones as either long or short, and were told to ignore the slightly higher pitch of the “deviant” ~10% of tones. Data was recorded with a Biosemi amplifier from 254 active-reference electrodes^3^, at sampling rate 256 Hz and 24-bit A/D resolution. Out of 12 participants, two had multiple recordings due to experiment interruption (subjects three and seven), and these were discarded from analysis in order to simplify the demonstration code.

All data was downloaded to the same directory, and files (which are all named “eeg_recording_1.bdf”) were renamed to the form “sNN_eeg_1.bdf” (where NN is a two digit number from [01.12]), to facilitate programmatic loading. The same procedure was applied to channel location files. This approach enables the simpler form of data loading in CTAP, i.e., programmatically building a Matlab data structure to represent all files with a given extension in a given folder. Another approach is discussed below.

For this paper, CTAP was run on a laboratory PC with Intel Core i7-7700, 3.6Ghz processor, 32GB RAM, Windows 10 Enterprise operating system. Timings of each pipeline are reported in Results.

### 2.2. The CTAP system

CTAP was introduced in Cowley et al. (2017)^4^. For this paper, the relevant points are as follows:

- CTAP is based on Matlab (version 2016b or later) and EEGLAB, latest versions preferred.
- CTAP is not compatible with EEGLAB graphical user interface (GUI), and therefore is not found in the EEGLAB list of plugins.
- CTAP is operated via scripts called from the Matlab command line (either with or without the Matlab desktop GUI).
- Example scripts provided are designed to run without editing (i.e., reasonable default parameters are provided), but will always require at least specification of relative data location.
- Despite the above point, it is advised to always tune one's parameters to the task at hand.

### 2.3. Analysis management: basic CTAP pipeline

The basic pipeline, runctap_manu2_basic.m, is defined to load the HeadIT data and channel locations from a single directory and preprocess it. A post-processing function follows preprocessing, to extract and plot grand average ERPs of the standard and deviant tones in short- and long-tone conditions. An electrode location above the left super-lateral temporal lobe was used to calculate ERPs (A31 in the HeadIT montage, close to T7 in the 10/20 system). ERPs lasted ±1 s around the stimulus onset, were baseline corrected by the mean of the signal within −1…0 s, and smoothed using a moving average of one quarter of the sample rate.

**Figure 1:**
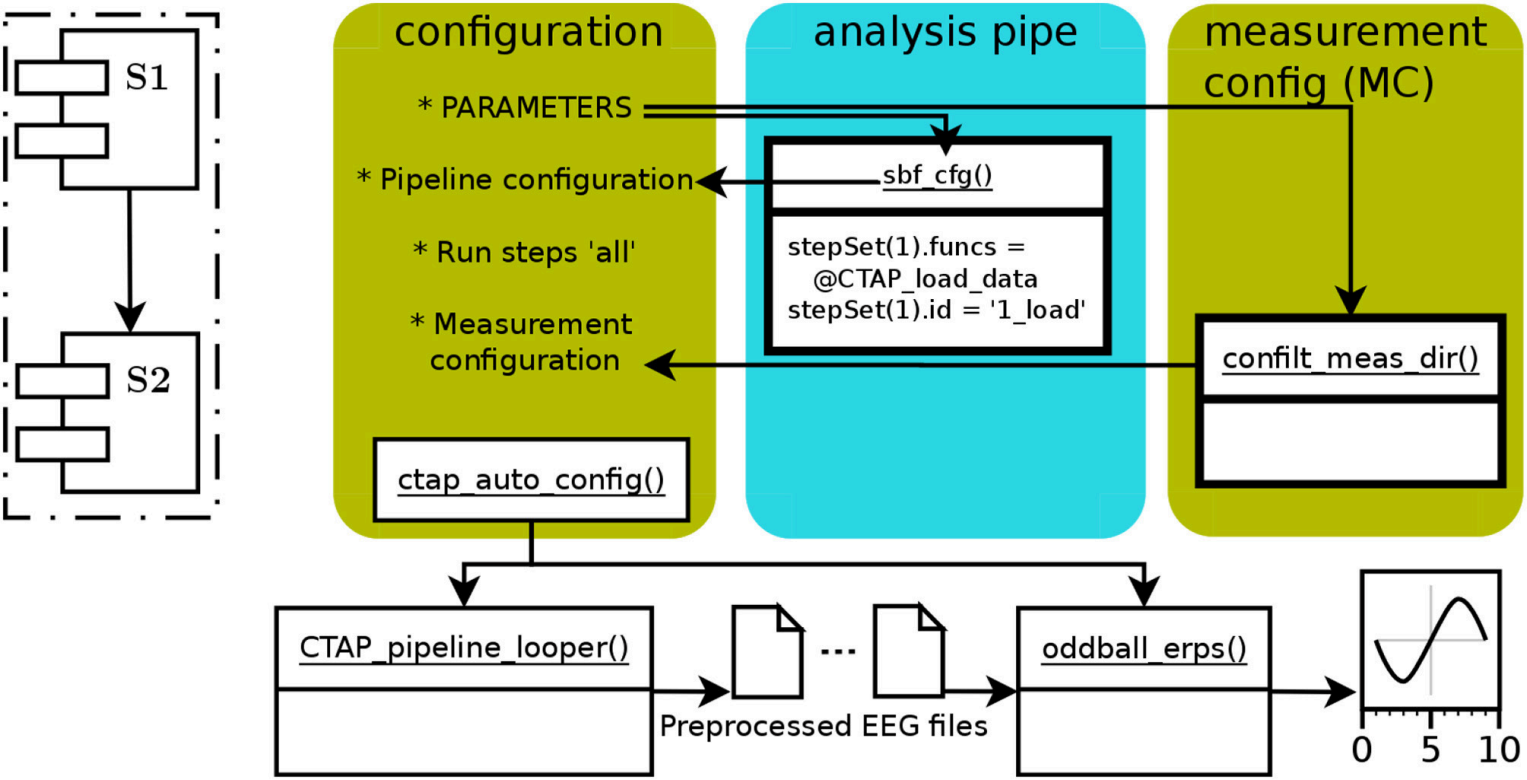
The schematic functional structure and data flow in the CTAP basic pipeline, showing the flow of data via arrows between functions (in white boxes) and scripted operations (marked by asterix). The colored, rounded-corner boxes show parts that a user must define (from left to right): (1) “configuration” box represents the main script with global parameters and calls to required functions. (2) the “analysis pipe” is defined in the “sbf_cfg()” subfunction, returning the Cfg structure. (3) the “measurement config” field of the Cfg structure, “Cfg.MC,” is obtained by scanning a given directory using “confilt_meas_dir().” Cfg is then processed by “ctap_auto_config()” and passed to “CTAP_pipeline_looper(),” directing the latter function from where to load and how to preprocess the data. The final output is then created by “oddball_erps().” The dashed-line inset shows a schema of the core operational process of the basic pipeline, consisting of two sequential step sets **S1**, **S2**; each containing multiple functions.

In Cowley et al. (2017), Figure 2 showed a schematic of the generic CTAP pipeline operative flow. Here, Figure 1 shows a similar schema for the basic pipeline. All CTAP pipelines are built around the “step set” structure, which is simply a list of function handles with an identifier string and possibly other control fields (e.g., save = true/false).

In runctap_manu2_basic.m, the first code section (lines 35–52) defines parameters that allow configuration of CTAP itself: usage variations can be obtained by, e.g., electing to overwrite old results, process subsets of subjects, or call subsets of the step sets.

The next section (lines 55–67) calls the necessary functions to create the key data structure Cfg. Initially, Cfg is defined in terms of functional steps, arguments to functions, and important fields such as preferred reference montage (e.g., average), labels of EOG channels to use for artifact detection, etc. Such code is wrapped in a sub-function sbf_cfg() for code readability, but can take any form. The subsequent lines are required to further specify which measurements to process and which steps to run. Finally Cfg is passed to ctap_auto_config() to check it is well-formed and has all required parts, and match arguments to functions.

The next two sections (lines 70–94) call the functional parts of CTAP: CTAP_pipeline_looper() preprocesses the EEG according to the step sets in Cfg; and ctap_manu2_oddball_erps() calculates and plots condition-wise ERPs. The step sets are simple. First, 1_load contains the steps to prepare data: loading, re-referencing, temporal blink classification, highpass filter, and Independent Components (ICs) Analysis (ICA) decomposition. The second step set, 2_artifact_correction, provides detection of ICs related to horizontal saccades (using ADJUST toolbox Mognon et al., 2011), and to blinks (using CTAP's built in method Cowley et al., 2017); then detection of bad channels by variance of the Median Absolute Deviation (MAD—of a channel from the dataset). Each detection routine is followed by a function that either rejects or corrects the bad data, and the bad channels are interpolated.

Step set 2 is sandwiched by taking two “peeks,” or snapshots, of the data state. The peeks serve to assess the state of the data before and after artifacts are removed, providing raw-data plots and statistical moments of segments of data which are synchronized between different points in the pipeline.

In addition to outputs that the user generates specifically via the pipeline (saved mainly as visual diagnostics from each function), CTAP stores the history of all operations and parameters in the EEG data structure. This history is also logged, showing in human-readable format all steps taken and their outcomes. Separate log files record all data rejected/corrected.

### 2.4. Output comparison: branched CTAP pipeline

**Figure 2:**
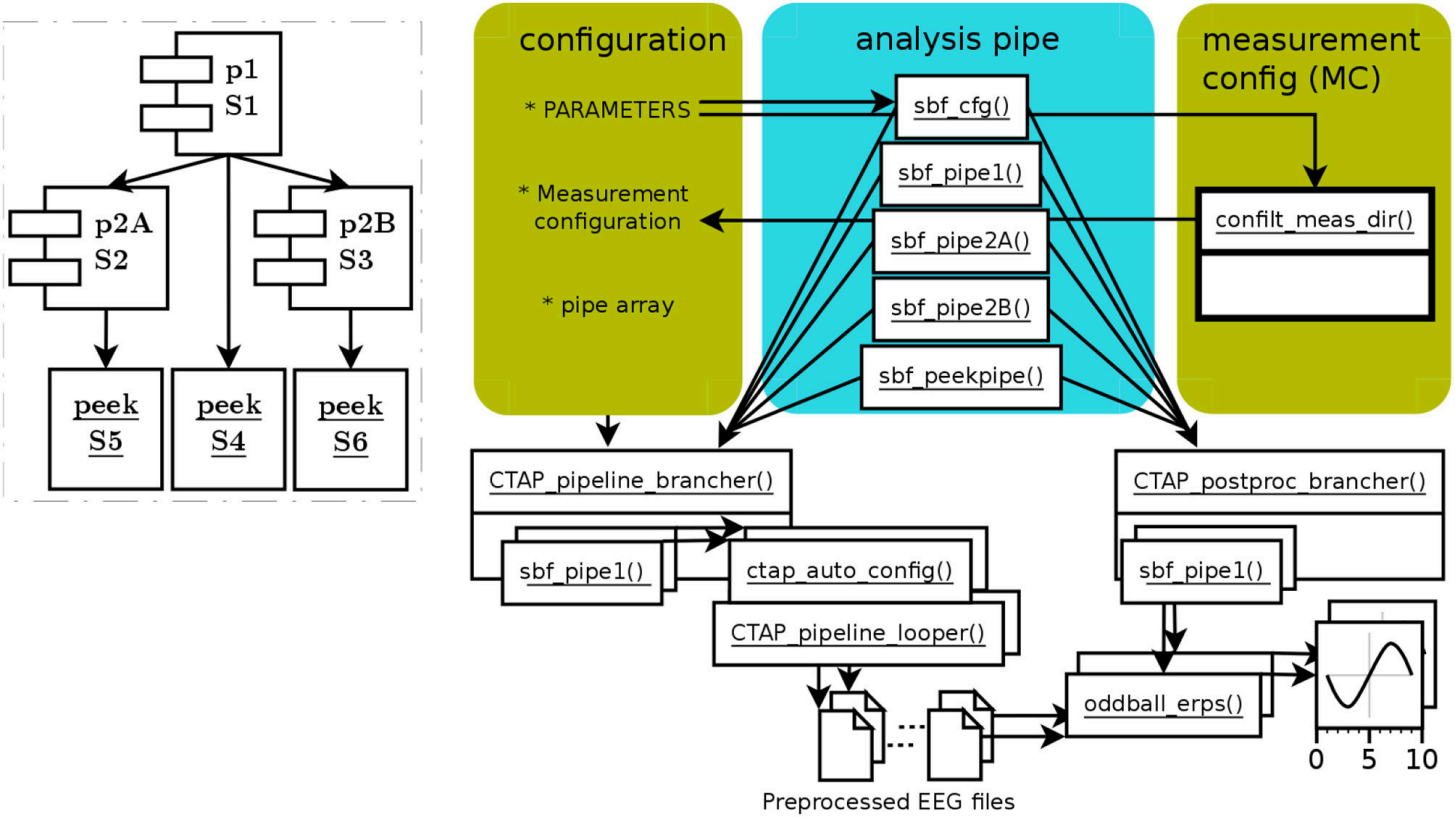
Schema of functions and data flow in CTAP branched pipeline. Scripted configuration has fewer specifications than in basic pipeline, because they are internal to pipe subfunctions. Each pipe subfunction is handled by CTAP_pipeline_brancher(), which configures the relationship to other pipes in terms of ordering and directories, and then invokes CTAP_pipeline_looper(). The same process can be repeated in CTAP_postproc_brancher(), calling a custom post-processing function on each “branch” of preprocessed data. The dashed-line inset again shows the core operational process, this time based on pipes (which here contain a single step set each). Despite the relative (compared to basic pipeline) complexity of the functional schema on the right, the inset shows the simplicity of the branched pipeline's topography.

The branched pipeline, runctap_manu2_branch.m, presents two alternative approaches to artifact detection (so represents the simplest form of branched tree). The code directly extends the basic pipeline, with the same step 1 and two alternative steps 2. Each step, 1, 2A, 2B, is encoded in a separate subfunction, which can be considered as separate *pipes*. Thus, pipe 1 is the “trunk” of the tree, and pipes 2A and 2B are two separate branches. These subfunctions are referenced in a cell array of function handles, which is passed to CTAP_pipeline_brancher() to process all requested parts. This brancher function loops through each pipe, and handles path creation and validating the Cfg structure. Figure 2 shows the structure of the branching pipeline.

The steps in pipes 1 and 2A replicate the steps in the basic pipeline. In order to illustrate parameter usage, we experiment with a small tweak of the parameters for bad channel detection in 2A, to try improving the noise rejection. The method “variance” is retained, but the MAD value is tweaked by the parameter “bounds” (a tuple representing lower and higher MAD at [−5;2.5]), and the outcome is constrained to the worst 5% (12) channels. Another pipe, 2B, is added for comparison. 2B attempts to do a general artifact detection over ICs, using methods from the FASTER toolbox (Nolan et al., 2010); and bad channel detection using the spectral method in EEGLAB.

In general usage, the branching approach changes from the linear approach as follows:

- step sets **must** be defined in subfunctions, so that they can become function handles in the pipe array;
- each pipe (after the trunk) must define one or more source pipes, from which data will be loaded, where source = pipe_ID#step_ID;
- each pipe must itself define the steps to run;
- when multiple calls to a single function are defined in an unbranched pipe, such as CTAP_peek_data() in the basic pipe, then arguments to that function can be declared just once (if they do not change across calls). In contrast, for branched pipes each pipe must declare arguments for its functions separately;
- as in the basic pipe, an ID is needed: here the ID is created inside each pipe, for clarity.

#### 2.4.1. Comparing branches

The branched pipeline creates similar outputs to the basic pipeline, but also provides the opportunity to compare branches. This can take two forms: comparisons between changes to data, and between data after change.

Comparing data after change is primarily done via the function CTAP_peek_data(). This function provides many options (documented in function help comments, Cowley et al., 2017, and the CTAP wiki) to output visual and statistical summary data from segments of EEG data selected at given or random time points. CTAP_peek_data() can also save the EEG and ICA-activation data within the peek segment (not set by default). Thus peeks can help compare between the outputs of different pipes. For numerical data (statistics or EEG/IC data), this process can be automated using the CTAP_postproc_brancher() function and simply taking the difference between earlier and later outputs. Reduction in range, skewness, kurtosis, for example, would all tend to indicate an improvement in signal to noise ratio (SNR).

In runctap_manu2_branch.m pipes are compared with a single final “peekpipe.” Peekpipe is given multiple source pipes: 1, 2A, and 2B; and will thus create multiple output directories, which are automatically labeled by the concatenated pipe ID and source ID. Peekpipe contains one step set with one function, CTAP_peek_data(). In this case, we set the step set “save” field to false, because the data is not meaningfully changed.

Changes by rejection of bad data are recorded in .mat file format in the pipe's “quality_control” directory, and can therefore be directly compared in Matlab. If changes to the data are of similar kind, e.g., rejection of bad channel artifacts by two separate methods, then the simple principle of parsimony can apply: given final data of similar quality, the method that rejects the least data should be preferred.

### 2.5. Parameter optimization: HYDRA pipeline

**Figure 3:**
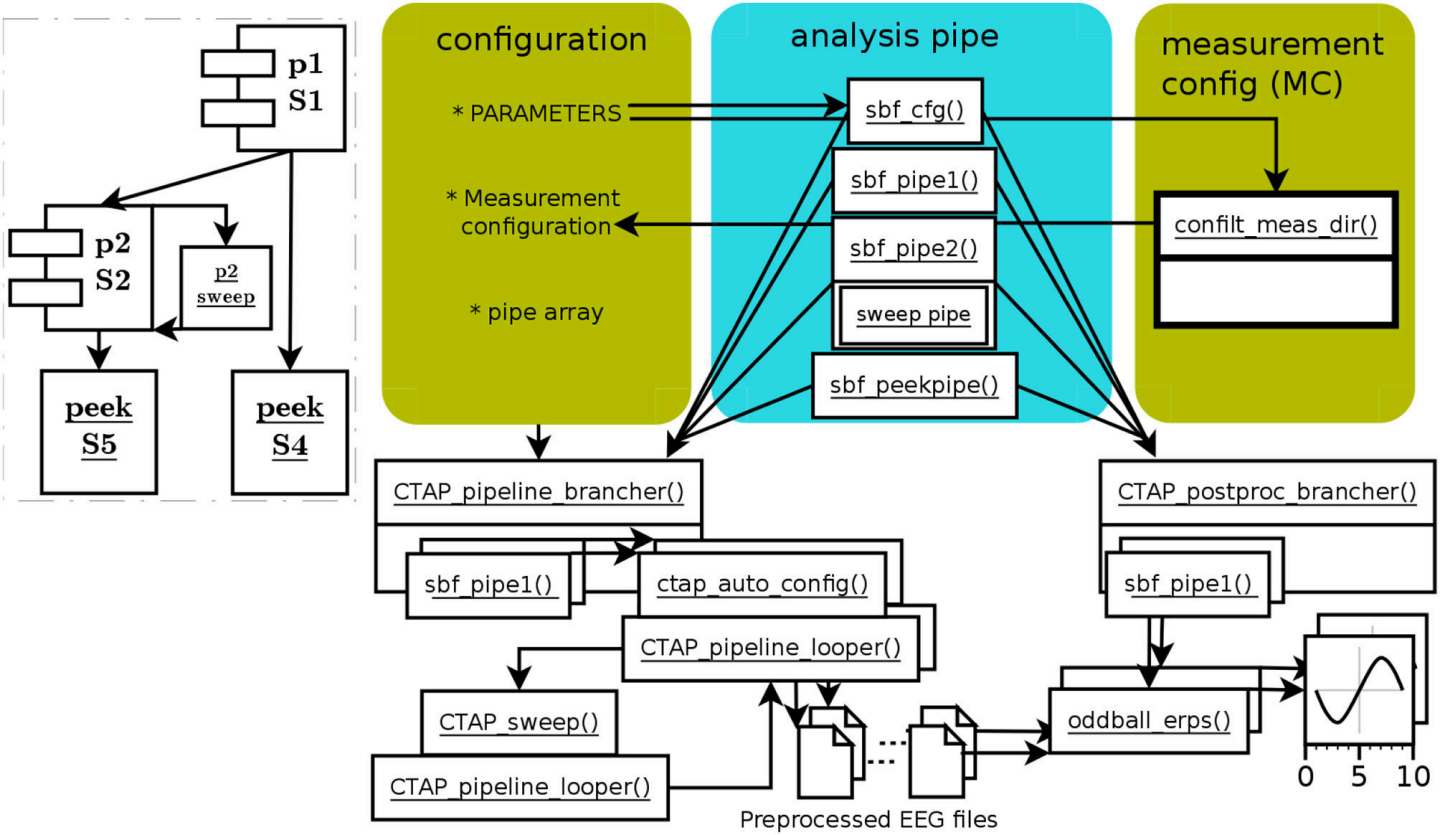
Schema of functions and data flow in HYDRA parameter-optimization pipe, based on the branched pipe. The branch 2B has been left out, and branch 2 defines a sweep step instead. The function CTAP_sweep() executes a given “mini-pipe” in a separate instance of CTAP_pipeline_looper() for each value in the given parameter range. The consequent range of outcome values (in terms of bad channels, ICs, segments, or epochs) is used to select a final value for the optimized parameter according to a simple criterion function. The dashed-line inset shows the relatively simple sweep process.

The HYDRA pipeline operates exactly as other pipelines, depending on which type it is based on: here, the branched pipeline. What distinguishes HYDRA pipelines is the inclusion of the function CTAP_sweep(), which attempts to find the optimal value from a range, for a given parameter of a target function. It does this by repeatedly testing the data against each value in the given range or set. The optimal value, selected by an objective function, is then passed back to the calling pipe to serve as the parameter value for a later call to the given function. This is all illustrated by the example pipeline runctap_manu2_hydra.m. Figure 3 shows the schematic structure of the HYDRA pipeline.

In this pipeline, the pipes 1 and 2 are similar to runctap_manu2_branch.m pipes 1 and 2A. Pipe 2 includes one extra function, CTAP_sweep(), placed right before its target function, CTAP_detect_bad_channels(). Thus the sweeping deals with data at that point, right after two types of bad component detection and handling. As well as the name of the function it will target, CTAP_sweep() takes as arguments the target function; the method for detection, variance; the parameter to sweep, “bounds”; and the range of values to sweep. Here, the parameter bounds is in the range 1 to 6 MAD, incrementing by steps of 0.2. This MAD range was chosen by empirical observation, noting that the extreme values result in either very few or very many channels rejected.

CTAP_sweep() also takes a “mini-pipe” parameter, SWPipe, which defines a step set that tests each value. CTAP_sweep(), by default, selects a final parameter value based on an estimate of the inflection point in the curve of outcome values. This point is defined here as the parameter value for which the change in outcome value from step *i* to *i+1* is closest to 1 standard deviation (SD) of the range of outcomes (where *i* is in [1.n-1], n = size of parameter range). The approach of selecting the parameter value by calculating the inflection point is chosen as a simple way to express a change in the signal which is big enough (i.e., not merely a fluctuation in the “plateau”), but not too big (i.e., following the steepest period of change).

The final value selected by CTAP_sweep() is passed back to pipe 2, becoming the “bounds” parameter for the subsequent call to CTAP_detect_bad_channels().

## 3. Results

Having run the pipelines on the HeadIT data, the user will be able to access extensive results as follows.

### 3.1. Basic CTAP pipeline outcome

Basic pipe completed preprocessing for each subject/EEG recording in ~40 min, on average. ERP creation then took ~270 s per subject.

The various human- and computer-readable bookkeeping performed by CTAP is documented, in Cowley et al. (2017) and on the repository wiki. The basic pipeline saves informative logs and quality control reports in the output directory. For example, the file logs/all_rejections.txt shows (after extracting suitable comparisons) that artifact routines removed ~11% of bad components, and ~5% of bad channels. The file logs/peek_stats_log.xlsx indicates that the minimum-to-maximum range and SD are both reduced by about 40%, to a between-subjects average value of 3,611 and 33 μV, respectively.

The peek stats included a Kolmogorov-Smirnov test, which indicated that every channel was approximately normally distributed. Thus, we can estimate that ~95% of the data lies within 2 SDs of the mean, which equates to a group average data spread of 136 μV (reasonable for EEG data in an ERP-analysis context).

The 40% reduction in data magnitude suggests that significant artifact removal occurred. The final range (>3.5 V), however, is higher than expected from neural sources, suggesting some artifact remained. The effect of time-locked signal averaging will enhance time-locked activations, diminish non-systematic noise, and reduce amplitude overall. Thus, we can look at ERPs to discover whether systemic noise is reduced to acceptable levels.

**Figure 4:**
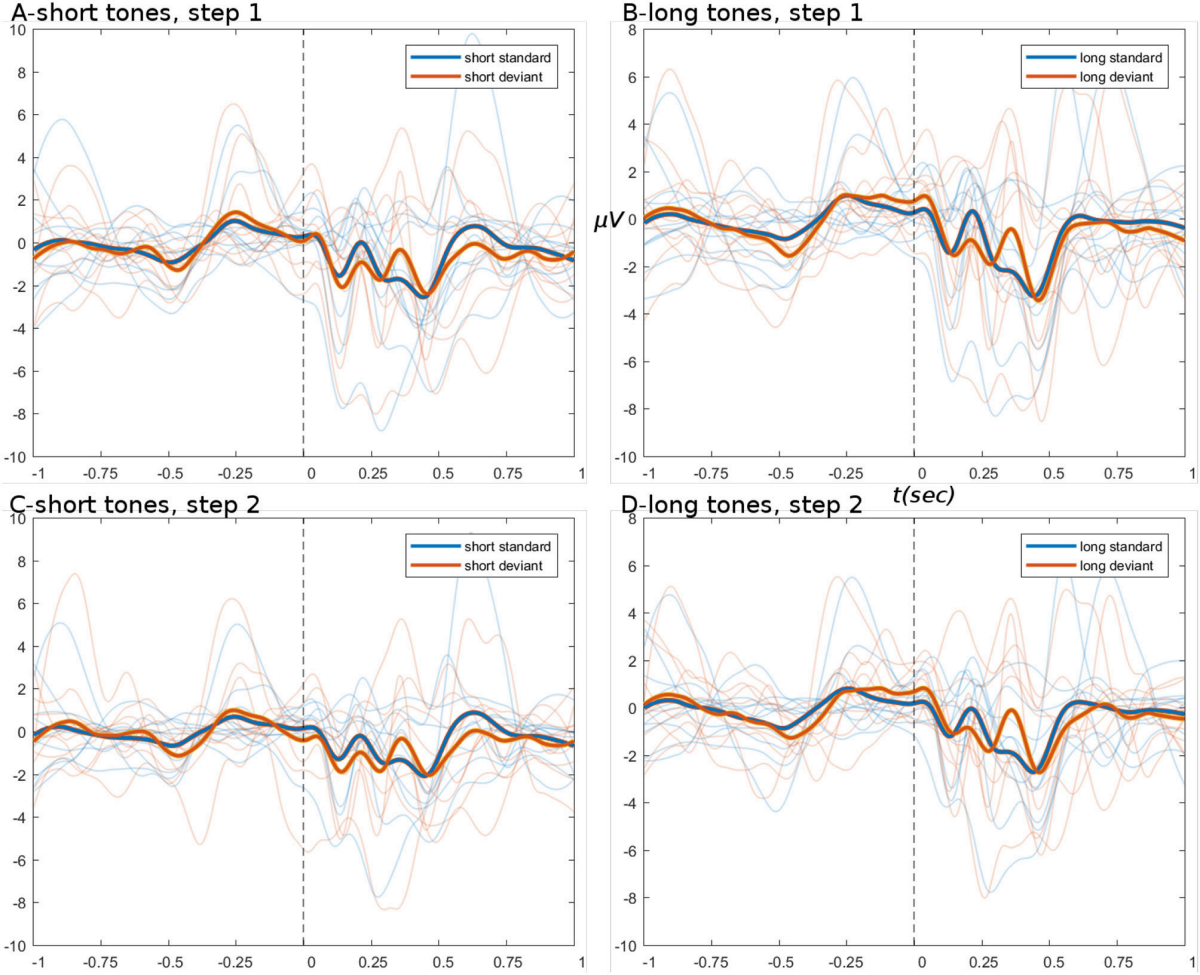
ERPs from basic pipeline data. **(A,B)** Show the short and long tone conditions, respectively, for data after step set 1. **(C,D)** Show short and long tone ERPs after step set 2. In all ERPs, blue colors are standard trials and red colors are deviant trials. Two thick foreground lines show the grand average, while per-subject averages are shown by narrow lines in background, illustrating group variance. Ordinate and abscissa are μVolts and time in seconds, respectively. Zero time marks the stimulus onset. Deviant trials show greater responding at peak P300.

The ERPs derived from the basic pipeline are shown below in Figure 4, for the data after step set 1 “initial loading” (top row), and after step set 2 “artifact correction” (bottom row). These ERPs show that little systematic change was induced by artifact correction, i.e., the upper (Figures 4A,B) and lower (Figures 4C,D) panels have the same amplitude ranges and very similar morphology. ERPs show an expected deviant-trial activation difference, especially at peak P300.

Examining the peek statistics by subject, it is clear that subject 12 is an outlier, with values an order of magnitude greater than any other subject, both before and after artifact correction (nevertheless, correction reduced range and SD by 43%). An experimenter could thus choose to remove this subject and proceed with the remaining data with good confidence. On the other hand, we can also examine this subject's CTAP output in more detail to identify why the data was not cleaned more, and determine how to improve results.

CTAP artifact detection steps illustrate their outcomes with visuals saved subject-by-subject under the quality_control directory. For the basic pipeline, this includes bad IC detection by ADJUST toolbox (Mognon et al., 2011), blink detection by CTAP's template method (Cowley et al., 2017), and bad channel detection by channel variance.

First, a scalp map plot shows at a glance the spatial activations of all the ICs detected by ADJUST: for subject 12 they all appear to be genuine artifacts. Check here:

quality_control/CTAP_detect_bad_comps/set2_fun2/

We can double check this by studying spectra and ERP-image for each IC, under:

quality_control/CTAP_reject_data/set2_fun3-badcomps/s12_eeg_1_session_meas/

Second, a scalp map plot shows spatial activations of any ICs identified as blink related: for subject 12 this appears quite blink–like. Check:

quality_control/CTAP_detect_bad_comps/set2_fun4/

We can then check the spectra and ERP-image of this IC: however the ERP-image (labeled “Continuous data”) does not show the characteristic pattern of a blink IC (short strong bursts of activation in an otherwise quiet signal). See:

quality_control/CTAP_filter_blink_ica/set2_fun5-blinkICs/s12_eeg_1_session_meas/

We can then examine the raw data ERP of vertical EOG and vertex channels, which shows no change from before to after correction (by filtering). Check here:

quality_control/CTAP_filter_blink_ica/set2_fun5-blinkERP/

**Figure 5:**
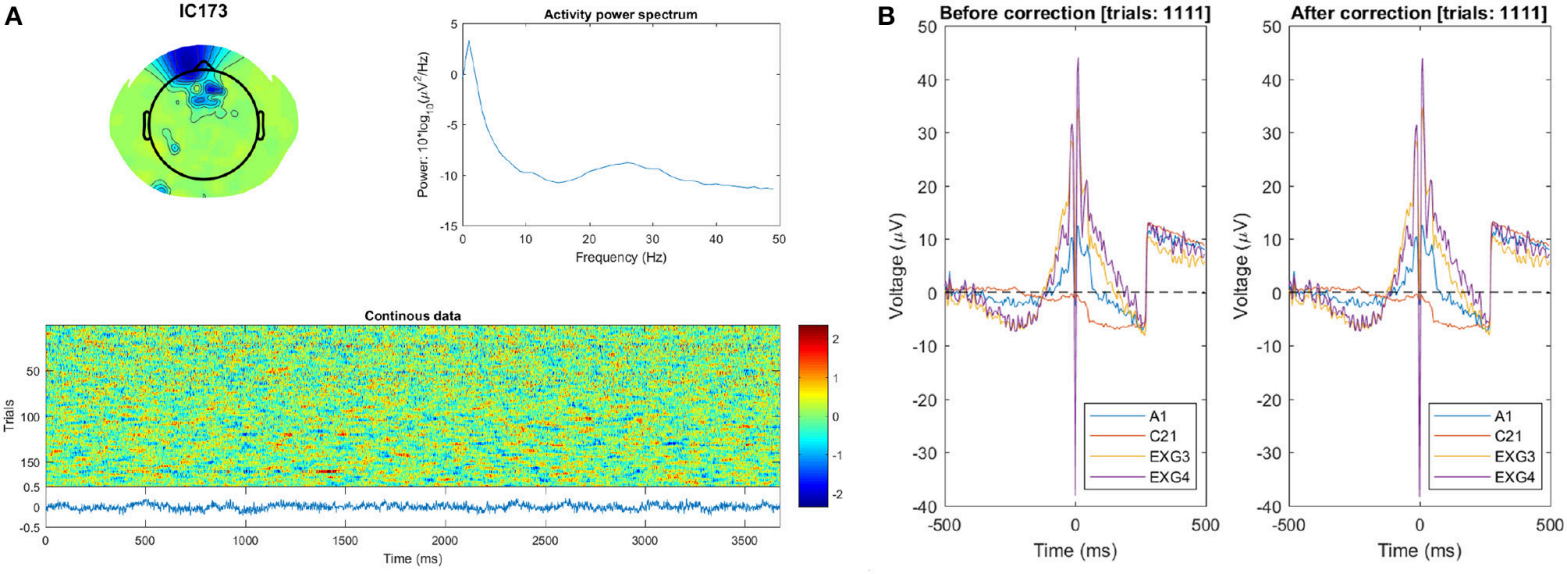
Subject 12 blink detection quality-control visuals. **(A)** The scalp map (top left), power spectrum (top right), and “Continuous data” ERP-image (below) of the IC detected as blink–related. The fact that the ERP-image does not contain blink–like activations especially suggests that selection of this IC (as a blink template) was a false positive. **(B)** ERP plot of the raw data during identified blink events, for vertical EOG and vertex channels. The ERP is clearly unchanged after correction (and very artifactual besides).

This suggests that blink–related activations remained in the data which could explain its large final magnitude. Blink-detection visualizations are shown in Figure 5.

Bad channel detection is the last step to check, starting with a histogram of channel variance values. See:

quality_control/CTAP_detect_bad_channels/set2_fun6-variance/

For subject 12, a subset of channels has variance lying far outside the threshold, while the rest are grouped near the median.

The function CTAP_reject_data() records the bad channels' scalp location and a raw data snapshot. See:

quality_control/CTAP_reject_data/set2_fun7-badchans/s12_eeg_1_session_meas/

The scalp map shows that all bad channels are located in the frontal scalp region, and thus probably dominated by ocular artifacts, which seems supported by the corresponding raw data. The co-located grouping of channels implies that interpolation from neighboring channels cannot provide a solution, and this recording cannot be used or processed further without solving the ocular artifact problem.

From these outputs, we can conclude that the pre-processing of (at least) subject 12 failed, but the failure was due to cascading effects of faulty blink removal, and thus the data might be salvageable with another approach. CTAP's branching functionality helps to more easily compare approaches.

### 3.2. Branched pipeline outcome

For each subject / EEG recording, branched pipe completed pipe 1 in ~25 min; pipe 2A in ~01:45; pipe 2B in ~04:50; and the peek pipe in ~3 min.

**Figure 6:**
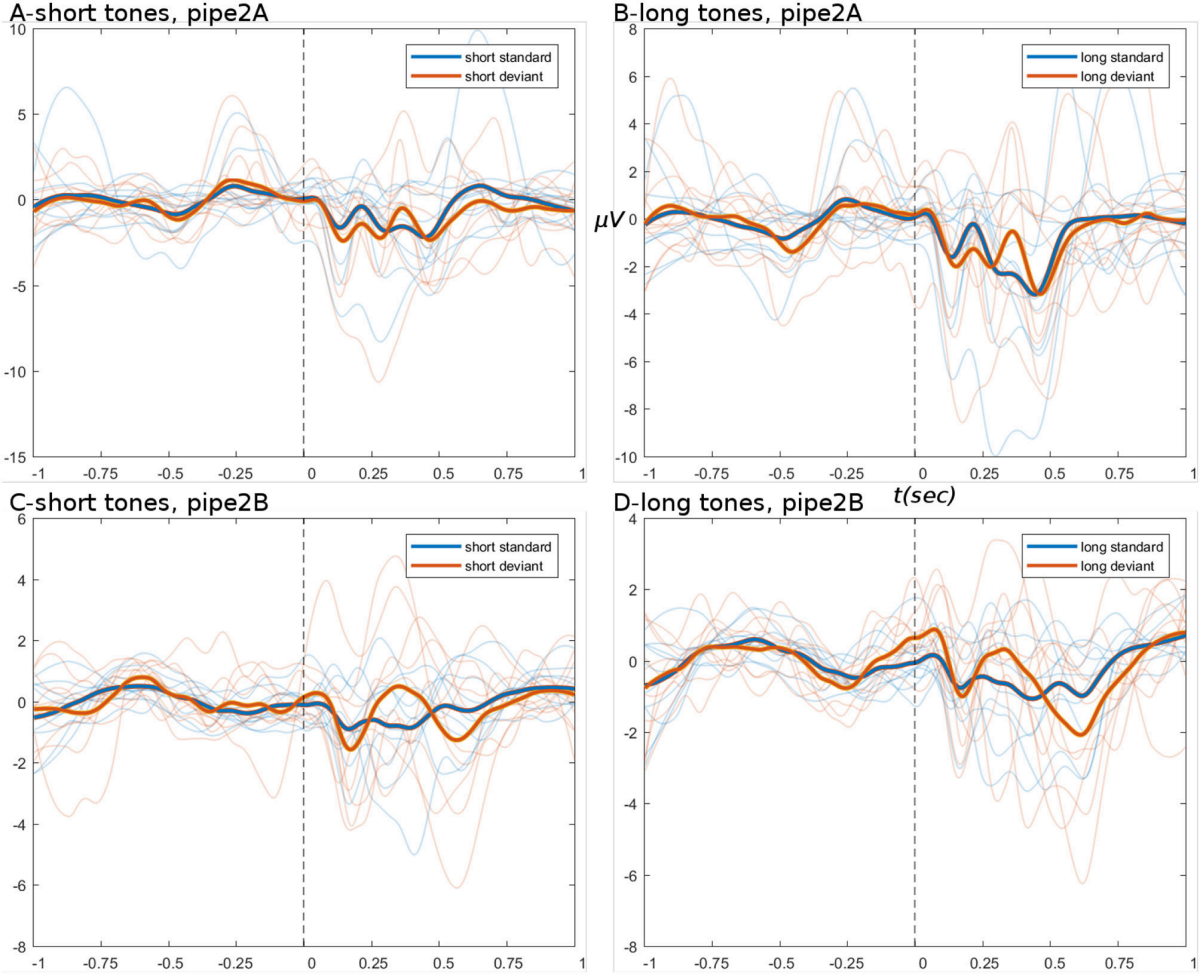
ERPs from branch pipeline data. **(A–D)** Show short and long tone conditions, respectively. **(A,B)** Show pipe 2A data; **(C,D)** show 2B. In all ERPs, display settings are as for Figure 4. ERPs show that pipe 2B provides greater noise-reduction than pipe 2A, particularly in the pre-stimulus period.

ERPs derived from the branched pipeline are shown in Figure 6, for the data after pipes 2A and 2B (data after pipe 1 are identical to basic pipe, step set 1). The outcome of pipe 2A is again similar to basic pipeline steps 1 and 2: the tweak of bad channel parameters had no significant effect, except to raise the channel rejection rate to 7% (bad ICs remained ~11%). The lack of effect might be explained by the observations regarding ocular artifacts in basic pipeline.

The outcome of pipe 2B is more productive: both short and long tone conditions show reductions in amplitude of 30 and 33%; while both conditions show greatly reduced variance of subject-wise averages in the pre-stimulus period. This followed data rejection rates of ~10% for bad ICs (by FASTER Nolan et al., 2010) and ~2% for bad channels (by EEGLAB's spectral method). The reduction in channel rejection rates (5 → 2%), while retaining good outcomes, may indicate improved specificity in bad IC detection. In pipe 2B, the FASTER toolbox is the primary means of removing troublesome artifacts that are temporally and spatially limited but still frequent enough to show in an ERP. The outcome of FASTER is visible in the scalp maps of detected bad ICs; see:

quality_control/CTAP_detect_bad_comps/set3_fun1/

**Figure 7:**
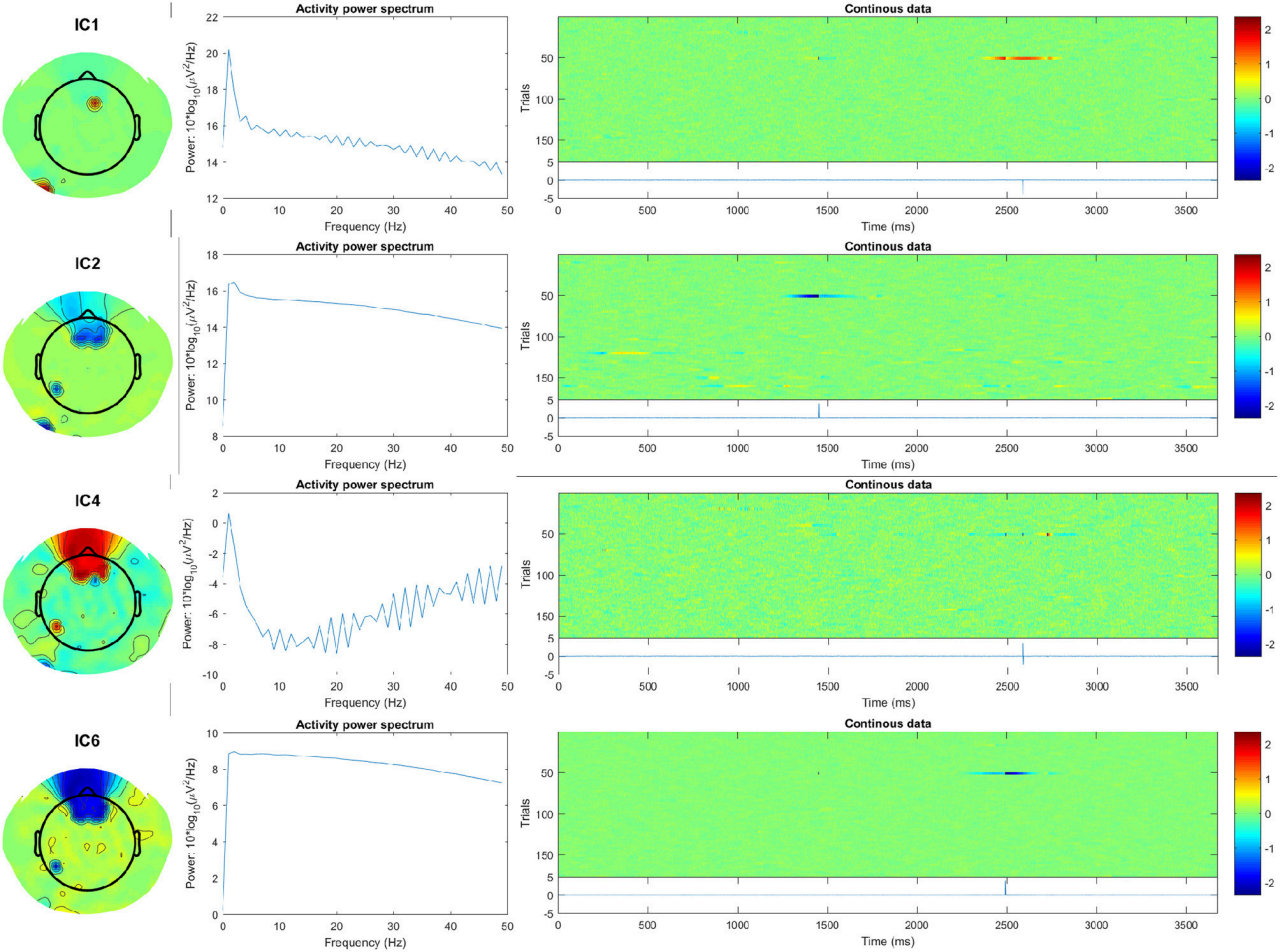
First four artifact ICs detected by FASTER for subject 12, showing scalp map, spectra, and ERP-image plots. The ERP (blue line), shown below the plot labeled “Continuous data,” indicates the temporally-isolated sharp impulse signals. These are likely to originate from non-biological artifact.

Here, subject 12 for example shows at least 10 strongly-activating frontally-located ICs. Examining the spectra and ERP-image plots of rejected ICs, we can see the first four ICs correspond to temporally- and spatially-isolated single-impulse signals of relatively large amplitude. See Figure 7, and compare with the outputs obtained after running the pipeline:

quality_control/CTAP_reject_data/set3_fun2_badcomps/s12_eeg_1_session_meas/

These components were not evident in the ICs detected by pipe 2A, probably because (a) the ADJUST toolbox was restricted to look for horizontal saccade-type ICs, and (b) the large activations are not blink–related and so would not be caught by CTAP's blink-template method. Overall, FASTER was programmed to be more liberal by setting the parameter match_logic = @any, meaning the detection function would trigger for any of FASTER's inbuilt metrics. Despite this, less bad ICs were detected for subject 12 using FASTER (25) than using ADJUST + blink-template (32+1).

Finally, no bad channels were detected for subject 12 after rejection of bad ICs detected by FASTER. This does not seem to be a mere failure of the spectral method, because (a) bad channels were detected for all other subjects, proving the method does work for this data; and (b) peek outputs for subject 12 do not show any clearly artifactual channels in either raw data or IC activations. See the peek at:

quality_control/CTAP_peek_data/set4_fun1/s12_eeg_1_session_meas/

This implies that the main problem with subject 12 was neither ocular nor channel artifacts, but strong impulse signals in frontal scalp locations (possibly the subject touched the electrodes).

### 3.3. HYDRA pipeline outcome

For each subject / EEG recording, HYDRA pipe completed pipe 1 in ~24 min; pipe 2 in ~10 min; and the peek pipe again in ~3 min.

**Figure 8:**
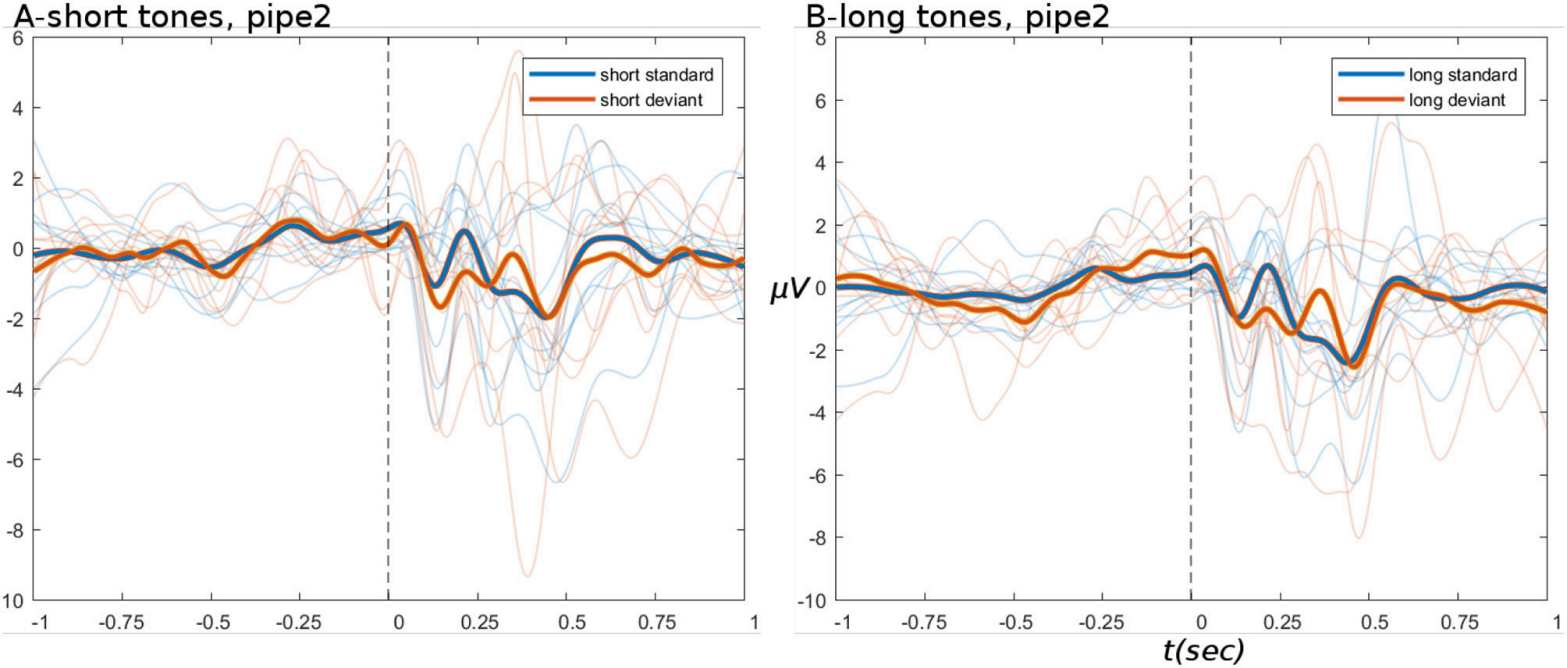
ERPs from HYDRA pipeline data. **(A,B)** Show short and long tone conditions, respectively. In all ERPs, display settings are as for Figure 4.

ERPs derived from the HYDRA pipeline are shown in Figure 8, for the data after pipe 2. These ERPs are of comparable quality to branched pipe 2B, especially in the pre-stimulus period.

The pipeline log logs/all_rejections.txt indicates that 10% of ICs (unchanged from branched pipe 2B) and 21% of channels were marked as bad, which is quite a large proportion. On the other hand, the large number of recorded channels implies that even losing a large fraction of them would not be catastrophic, so long as the bad channels were spatially distributed widely across the scalp. This can be determined from the scalp maps saved when channels are rejected, see:

quality_control/CTAP_reject_data/set2_fun5_badchans/

Unfortunately, the example subject 12 returned 88/~34% bad channels, many clustered around the frontal scalp area. This suggests that (a) bad channel detection by variance may be a non-optimal method in this case; and (b) the method for selecting the final parameter value in the sweeping function may be too greedy.

**Figure 9:**
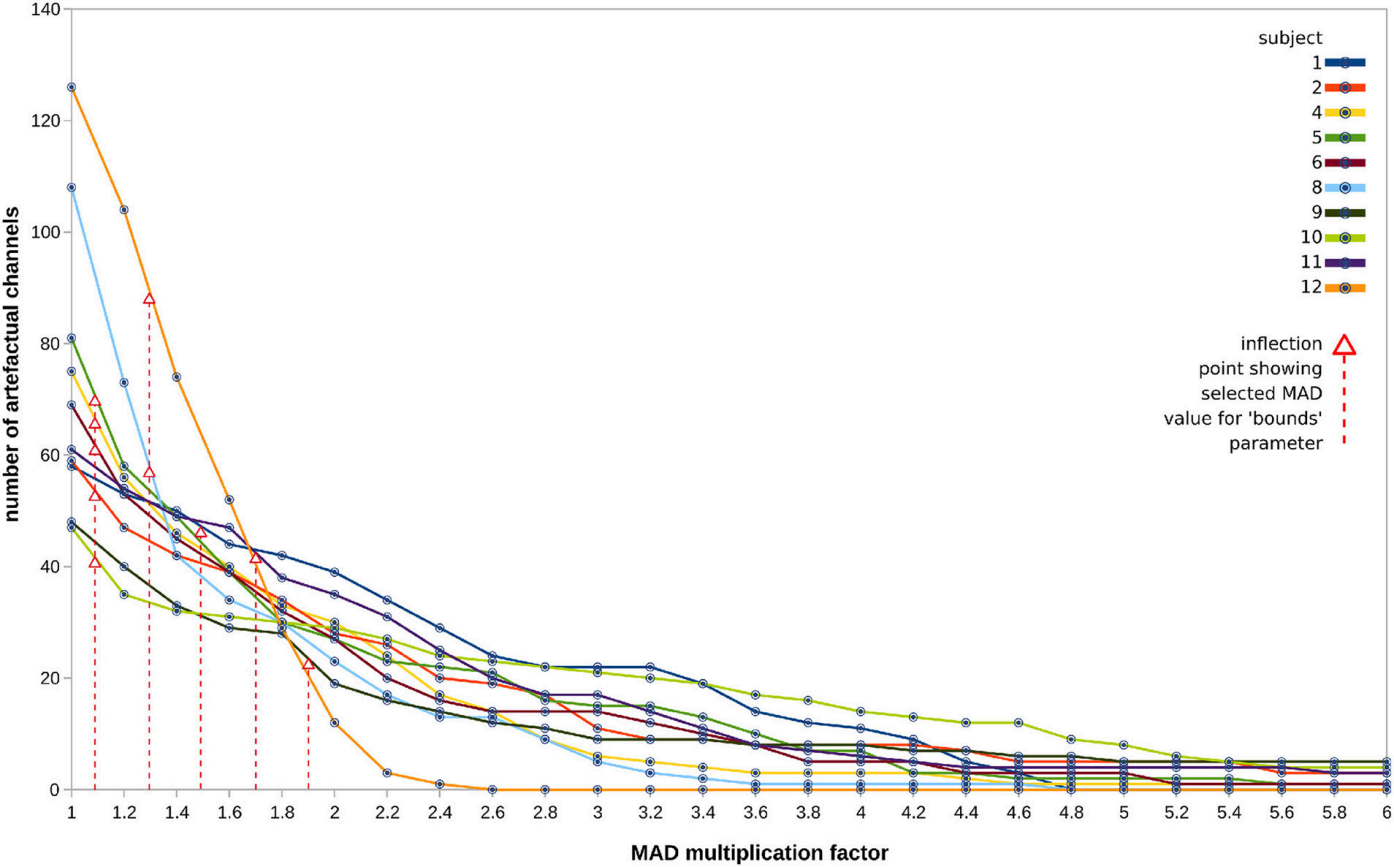
Sweep outputs for all 10 subjects (one line graph per subject), in terms of number of bad channels classified per each value of MAD in the range 1.0–6.0, in steps of 0.2. Discovered “inflection points,” marked by red arrows, were used to derive the MAD value chosen as the final parameter, indicated with vertical red lines.

The sweeps detected a number of artifactual channels which tended to follow an expected exponential decay, as shown in Figure 9. The discovered “inflection points” (shown in Figure 9 as red arrows) mark the MAD value which was then chosen as the final parameter to pass to the pipeline. The selected values tend to be quite low in the range, which seems problematic for cases with rapidly falling numbers of detected channels. More robust methods under development are discussed below.

## 4. Discussion

Comparison of all pipeline outputs suggests that branched pipe 2B gives best performance, based on IC detection by FASTER toolbox, and channel detection based on spectral profile. Pipe 2B appears to provide the best distinction between standard and deviant conditions at the key P300 component, for both long and short tones. It is outside the scope of this paper to assess the neurocognitive results themselves; however these ERPs clearly show the morphology we should expect from an oddball task, with the “novelty-processing” P300 strongly responding to the deviant tone.

The strong performance of pipe 2B supports an approach based on bad IC detection via multiple features, as exemplified by the FASTER toolbox (see Figure 6), but it is dependent on the very high-spatial resolution dataset involved. Having many channels implies many ICs and thus it is worth searching across more feature spaces—FASTER by default searches five (Nolan et al., 2010). The outcome is higher sensitivity to artifacts, at the possible cost of specificity, but the cost can be borne due to the large number of ICs. Pipe 2B also compares well with HYDRA pipe 2 (Figure 8), which used FASTER, suggesting that spectral detection of bad channels outperforms variance-based detection, even when the variance threshold is not fixed but selected recording-by-recording ^5^. The “rejspec” method is also based on a fixed threshold, which could be selected per recording in a data-driven manner; however this approach was not chosen because “rejspec” is very slow.

Indeed, the methods selected were not chosen because they would be optimal, but rather because they complete in a reasonable time on regular computing hardware (not high-performance). Also, we chose well-known and understood methods, to permit the reader to focus on the novel elements we introduce.

The first, basic pipeline shows how CTAP meets the first three SWMS criteria: (1) replicable, (2) traceable, (3) self-monitoring.

First, the pipeline code completely encapsulates the processing: there are no vaguely-defined or manual steps, and configuration and running of the pipe are quite separate, which supports transparency and documentation. In addition, if the base CTAP code against which the pipe is defined should change, the EEG files preprocessed by CTAP will still contain detailed descriptions of the history of operations and complete parameter values. These factors make CTAP workflows *replicable*, meeting the first criterion of Saxena and Dubey (2011).

Second, the data provenance is encapsulated in the measurement configuration structure. In the demonstrated approach, the measurement structure is built merely by passing the input directory to confilt_meas_dir(). It is also possible to use a more robust (but more effortful) method, by defining a spreadsheet of subjects, recordings, and associated data. Using this approach, data can be stored in any directory structure desired, e.g., users might wish to store EEG recordings alongside clinical data in per-subject directories. The measurement configuration options make CTAP workflows *traceable*.

Third, the core CTAP looper is designed to allow pipes to experience errors without crashing the batch, i.e., errors will be logged and the currently-executing file will not complete, but later files will be processed as normal. On the other hand, while in development, the combination of debug mode and step sets allows for fine-grained examination of process outputs. A pipe can match steps into sets with any frequency, from one set for all functions, to one set per step. Thus, data can be saved for examination after all functions, after each function, or any combination. Thus, CTAP workflows are to a degree *self-monitoring*.

Branched CTAP workflows meet the fourth SWMS criterion: they are *configurable/scalable*. The branched pipeline begins to show the potential of CTAP. Only a single function is required to contain the peekpipe. Yet when applied with every other pipe as a source, the result is a tree of six nodes, which (for minimal programming effort) offers comparison of both parallel *and* sequential stages. Comparison between parallel nodes can be interesting (i.e., 2A vs. 2B), but it is more interesting to examine the evolution of data, e.g., applying some summary functions to sequential nodes to track data distribution statistics over time. Though the example tree shown in this paper is rather simple, it hints at the many possibilities available. For example, the multi-source feature could allow, not just a single pipe as above, but whole tree to branch off of every node in an existing tree. This could be used, e.g., to generate competing ERP-derivation approaches from multiple levels of preprocessing, testing the effect of increased information removal on ERPs.

Finally, the HYDRA pipeline shows how CTAP meets the *data-driven* criterion. Obtaining a parameter value from repeated testing of the data at a given point implies that the pipeline becomes tuned to each specific recording from the point of sweeping onwards. Given that it is a completely replicable automated operation, it cannot be considered a case of cherry-picking. Although this part of CTAP is a work in progress (future work is described below), the ability to seamlessly blend parameter sweeping into an EEG processing workflow is novel.

The core structure of the workflow is Cfg, creation of which is one of the most important parts of CTAP, and is a combined effort of the user and CTAP. In the branched approach, users have great flexibility to define Cfg, since it can be generated by one or many functions and/or scripts, which each may contain self-modifying arguments such as source or runSet specification. On the other hand, certain arguments such as pipe ID and source ID (which are usually created inside each pipe for clarity), could alternatively be passed in as arguments. This would require more complex parameterization of various functions, but in return would allow more robust re-configuration of the workflow tree by changing sources.

The ERP function, designed specifically for this dataset, is very simple because showing such visuals is secondary to the main objective of showing workflow management features. In fact, a more comprehensive ERP analysis solution is under development for CTAP, as a package for the R statistics computing platform (R Development Core Team, 2008).

Extraction of features is another capability of CTAP that is of general interest for EEG work. This includes features such as oscillatory band power in predefined segments, and also file-wise meta-data that is normally accessible only when an EEG file is loaded in Matlab, which can be too slow for automated file-management purposes. Such features are not exported in the demonstration pipes for this paper because they are not central to SWMSs, and have been shown elsewhere (Cowley et al., 2017).

### 4.1. Limitations and future work

CTAP is still under development, and as such does not contain all planned/required functionality, nor guarantee stability of code. Indeed the HYDRA functionality is pending publication as a peer-reviewed article, and may undergo considerable change by that time.

For example, the method of selecting final values from a parameter sweep is a matter of on-going work. In the method used, inflection points represent the midway mark between two testing steps for which the difference in number of bad channels is close to 1SD of the whole set of tests. As such, the method is too sensitive to the length of the “tail.” It besides takes no account of important domain-specific considerations. For example for bad channel detection, the spatial distribution is important: channels should not be too clustered or they cannot be interpolated from their neighbors. In development is a method of selecting a final parameter for bad channel detection methods that trades off the number of bad channels with the uniformity of their spatial distribution.

Currently, HYDRA implements just a simple range sweep. Thus the choice of final value is blind, i.e., cannot account for the “ground-truth” of whether detected artifacts are true positives or false positives. An upgrade is in development utilizing synthetic data, extrapolated by auto-regression from the recorded EEG data at the point of sweeping, and injected with synthetic artifacts. This synthetic dataset resembles the original, but contains fully-known ground-truth, such that detection algorithm classifier performance can be assessed in terms of specificity and sensitivity. This upgrade is expected to be published within a year of this writing.

A major problem when aiming to standardize EEG processing is choosing the point of reference. It is well-known that the reference strongly affects both quality and interpretation of the signal. However references are usually chosen according to the custom of the field, as either a particular point on the head or an average of all electrodes, and results thus potentially quite arbitrary. Established techniques exist to standardize the reference electrode: for example Yao's (2001) method sets the reference as a point at infinity, effectively creating zero-reference data, with EEGLAB integration (Dong et al., 2017). The PREP pipeline also provides an approach to reference standardization (Bigdely-Shamlo et al., 2015). CTAP will integrate one or more of these options in the long run.

### 4.2. Conclusion

We described CTAP, a toolbox to provide the features of a SWMS, aiming to make EEG preprocessing more replicable and scalable in an era when very large EEG datasets are becoming a more routine reality. The article demonstrated processing for a genuine dataset, recorded in an experimental context and freely available online in perpetuity.

Three “modes” of CTAP use were demonstrated, each one building on the one before to expose more functional features that assist the user in managing their EEG workflow. Although many of these features are by themselves quite minor, in combination they provide the basis of a flexible SWMS for EEG preprocessing.

CTAP is currently beta software, but is already used in several research and clinical sites across the Nordic region, processing from tens to tens of thousands of EEG recordings. Development is ongoing, and further integration of CTAP with other functionality from the EEGLAB ecosystem is expected to improve the overall usefulness and usability of all components: the whole becoming greater than the sum of its parts.

## Ethics statement

This study uses a dataset obtained from an online repository of human electroencephalography datasets, whose administrators were responsible for the correct application of ethical procedures. The paper refers to the dataset but does not reproduce it or host it elsewhere than its original place of publication.

## Author contributions

BC conceived CTAP, the paper and the associated pipelines. JK designed core CTAP architecture. Both BC and JK developed CTAP codebase. BC performed the analyses and wrote the paper, and prepared figures. All authors contributed to manuscript revision, read and approved the submitted version.

### Conflict of interest statement

The authors declare that the research was conducted in the absence of any commercial or financial relationships that could be construed as a potential conflict of interest.

## Footnotes

^1^In this paper we discuss “recording setups” to refer to hardware, protocols and environment with which data was recorded; and “analysis pipelines” to refer to software/hardware the data is processed with.

^2^The page http://headit.ucsd.edu/studies/9d557882-a236-11e2-9420-0050563f2612 links to data with a complete description of each recording.

^3^Note, the channel locations DO NOT match the locations of the Biosemi 256 channel cap published on the Biosemi website. Only channel location files found on the HeadIT website should be used. Channel location settings used in this paper have been verified by correspondence with Julie Onton, one of the original experimenters at SCCN.

^4^And see documentation at http://github.com/bwrc/ctap/wiki – in this paper, the term “wiki” will refer to this URL.

^5^However, see Limitations below with respect to the method of selection, which is work in progress at the time of writing. In this respect, HYDRA is not *currently* a strong contribution in terms of computer science; but shows how CTAP provides a frame.
